# The co-development of a linguistic and culturally tailored tele-retinopathy screening intervention for immigrants living with diabetes from China and African-Caribbean countries in Ottawa, Canada

**DOI:** 10.1186/s12913-023-09329-3

**Published:** 2023-03-29

**Authors:** Valerie Umaefulam, Mackenzie Wilson, Marie Carole Boucher, Michael H. Brent, Maman Joyce Dogba, Olivia Drescher, Jeremy M. Grimshaw, Noah M. Ivers, John G. Lawrenson, Fabiana Lorencatto, David Maberley, Nicola McCleary, Sheena McHugh, Olivera Sutakovic, Kednapa Thavorn, Holly O. Witteman, Catherine Yu, Hao Cheng, Wei Han, Yu Hong, Balkissa Idrissa, Tina Leech, Joffré Malette, Isabelle Mongeon, Zawadi Mugisho, Marlyse Mbakop Nguebou, Sara Pabla, Siffan Rahman, Azaratou Samandoulougou, Hasina Visram, Richard You, Junqiang Zhao, Justin Presseau

**Affiliations:** 1 Clinical Epidemiology Program, Ottawa Hospital Research Institute, Ottawa, Canada; 2 Department of Ophthalmology, Maisonneuve-Rosemont Ophthalmology University Center, Université de Montréal, Montreal, QC Canada; 3 Donald K Johnson Eye Institute, University Health Network, Toronto, Canada; 4 Department of Ophthalmology and Vision Sciences, University of Toronto, Toronto, Canada; 5 Department of Family and Emergency Medicine, Université Laval, Québec, Canada; 6 Centre for Research On Sustainable Health, VITAM, Université Laval, Québec City, QC Canada; 7 Department of Medicine, University of Ottawa, Ottawa, Canada; 8 Women’s College Research Institute, Women’s College Hospital, Toronto, Canada; 9 Department of Family and Community Medicine, University of Toronto, Toronto, Canada; 10 School of Health & Psychological Sciences, City, University of London, London, UK; 11 Centre for Behaviour Change, University College London, London, UK; 12 Department of Ophthalmology, The Ottawa Hospital, Ottawa, Canada; 13 School of Epidemiology and Public Health, University of Ottawa, Ottawa, Canada; 14 School of Public Health, University College Cork, Cork, Ireland; 15 Division of Endocrinology & Metabolism, Faculty of Medicine, St. Michael’s Hospital, University of Toronto, Toronto, Canada; 16 Dalla Lana School of Public Health, University of Toronto, Toronto, Canada; 17 Patient Local Advisory Group, Ottawa, Canada; 18 Centretown Community Health Centre, Ottawa, Canada; 19 West Ottawa Specialty Care, Ottawa, Canada; 20 School of Nursing, University of Ottawa, Ottawa, Canada; 21 School of Psychology, University of Ottawa, Ottawa, Canada

**Keywords:** Diabetic Retinopathy, Retinal Screening, Tele-retinopathy, Health services, Intervention development, Theoretical Domains Framework, Patient Involvement, Patient oriented research, Stakeholder consultation

## Abstract

**Background:**

Diabetic retinopathy is a sight-threatening ocular complication of diabetes. Screening is an effective way to reduce severe complications, but screening attendance rates are often low, particularly for newcomers and immigrants to Canada and people from cultural and linguistic minority groups. Building on previous work, in partnership with patient and health system stakeholders, we co-developed a linguistically and culturally tailored tele-retinopathy screening intervention for people living with diabetes who recently immigrated to Canada from either China or African-Caribbean countries.

**Methods:**

Following an environmental scan of diabetes eye care pathways in Ottawa, we conducted co-development workshops using a nominal group technique to create and prioritize personas of individuals requiring screening and identify barriers to screening that each persona may face. Next, we used the Theoretical Domains Framework to categorize the barriers/enablers and then mapped these categories to potential evidence-informed behaviour change techniques. Finally with these techniques in mind, participants prioritized strategies and channels of delivery, developed intervention content, and clarified actions required by different actors to overcome anticipated intervention delivery barriers.

**Results:**

We carried out iterative co-development workshops with Mandarin and French-speaking individuals living with diabetes (i.e., patients in the community) who immigrated to Canada from China and African-Caribbean countries (*n* = 13), patient partners (*n* = 7), and health system partners (*n* = 6) recruited from community health centres in Ottawa. Patients in the community co-development workshops were conducted in Mandarin or French. Together, we prioritized five barriers to attending diabetic retinopathy screening: language (TDF Domains: *skills, social influences*), retinopathy familiarity (*knowledge, beliefs about consequences*), physician barriers regarding communication for screening (*social influences*), lack of publicity about screening (*knowledge, environmental context and resources*), and fitting screening around other activities (*environmental context and resources*). The resulting intervention included the following behaviour change techniques to address prioritized local barriers: *information about health consequence, providing instructions on how to attend screening, prompts/cues, adding objects to the environment, social support, and restructuring the social environment.* Operationalized delivery channels incorporated language support, pre-booking screening and sending reminders, social support via social media and community champions, and providing using flyers and videos as delivery channels.

**Conclusion:**

Working with intervention users and stakeholders, we co-developed a culturally and linguistically relevant tele-retinopathy intervention to address barriers to attending diabetic retinopathy screening and increase uptake among two under-served groups.

**Supplementary Information:**

The online version contains supplementary material available at 10.1186/s12913-023-09329-3.

## Background

Diabetic retinopathy is a leading cause of preventable blindness in working-aged Canadians [1] and worldwide [2, 3]. Retinopathy involves microvascular damage to the retina that leads to swelling of the central retina and abnormal blood vessel growth that can lead to vision loss if not detected early and treated [4]. Early diagnosis and treatment are effective in preventing vision loss associated with diabetes. Canadian clinical guidelines recommend yearly diabetic retinopathy screening (DRS) for people living with diabetes to reduce the risk and progression of vision loss [5]. Screening for diabetic retinopathy is one of the most effective and least costly ways to reduce severe complications associated with this condition [6]. However, diabetic retinopathy screening rates are low across Canada. For example, in a teleophthalmology project across 5 provinces in Canada, over 68% of the study’s cohort of individuals living with diabetes had not attended screening in the last year, and almost a third never had [7]. Furthermore, diabetic retinopathy screening rates are often lower among cultural and linguistic minority groups [8], and among newcomers to Canada, including people arriving from China, Africa, and the Caribbean; groups at higher risk of developing diabetes complications [4].

The 2021 Canadian census showed that 21.9% of the Canadian population were foreign-born, and recent newcomers to Canada represented 3.5% of the total population [9]. In the capital city of Canada (Ottawa), a 25% sample of census respondents showed that 17% residents had immigrated from Africa and 48% from Asia [10]. Those immigrating from Asia were predominantly from China, making up 17% of the population [10]. Linguistically, approximately 65% of the immigrant population’s mother tongue is Mandarin and 8% speak French as their mother tongue in Ottawa [11].

Tele-retinopathy screening is a potentially useful way to deliver and improve access to diabetes eye care [12, 13]. Tele-retinopathy screening involves capturing, securely transmitting, and remotely grading retinal digital images, and referring individuals living with diabetes by eye specialists for further care [14]. There is limited work about tele-retinopathy screening conducted in Canada amongst key subgroups with ethnocultural and linguistic minority groups. The present study builds on our foundational research and studies investigating barriers and enablers of DRS attendance among newcomers and immigrants to Canada from China (Mandarin-speaking) and African and Caribbean (French-speaking) countries [15]. Also, the current evidence base is relatively silent on interventions targeting specific population groups [16].

Our work has demonstrated that immigrants face specific barriers and enablers that likely need to be addressed to create culturally sensitive and effective screening programs for these groups. In a study conducted with newcomers and immigrants to Canada from China and African-Caribbean countries living with diabetes, several barriers were identified and prioritized to help these individuals get their eyes screened [15]. Some of these barriers included: access to retinopathy screening itself, language barriers, lack of knowledge about diabetic retinopathy, fears about screening harming eyes, and other barriers, including remembering to get eyes screened, lack of transparency about costs, and family and healthcare provider influences [15].

Lack of access to DRS is a clear barrier, and tele-retinopathy screening is a promising and cost-effective solution [17]. However, improving access and providing tele-retinopathy screening alone will not ensure newcomers and immigrants attend. While tele-retinopathy screening addresses access-related barriers, additional behaviour change and implementation strategies are needed to address other barriers related to the uptake of services. These strategies need to be co-developed with communities and the health services surrounding them and informed by which strategies have already been shown to be effective [18].

Our overarching aim was to co-develop, with patient and health system stakeholders, a linguistically- and culturally relevant tele-retinopathy screening intervention for immigrants to Canada from China and African-Caribbean countries. Here, we aim to describe the systematic development of an intervention to improve DRS attendance informed by theory, evidence and patient and stakeholder involvement.

## Methods

### Design

Our overall approach is largely consistent with O’Cathain et al.’s [19] broad taxonomy of approaches for developing interventions, which highlights eight categories of approaches to intervention development. Among the identified approaches, we partnered with those who will engage in the intervention; took a population centered approach of the views of those engaging in the intervention; used evidence and theory; prioritising real-world implementation; used a systematic development process; developed an approach tailored to the given intervention; and combined components into the intervention [19]. To operationalise these approaches, we combined a behaviour change theory-based approach to intervention development with a co-development process involving patients and healthcare system stakeholders. Our overarching theoretical approach was rooted in French et al.’s [20] process model for developing theory-based behaviour change interventions, i.e., Who, needs to do what, differently; identify barriers and enablers to be addressed; identify potential behaviour change techniques to overcome the barriers and enhance the enablers; and determine how behaviour change be measured and understood. Our co-development process was rooted in the Framework of User-Centred Design [21], which emphasizes iterative development with those for whom an intervention is developed and underscores three concepts: understand users, develop and refine intervention prototypes, and observe users’ interactions with the prototype. User-centred (human-centered) design is an umbrella term of many design approaches [22]. We sought to co-develop the intervention, sharing power and decisional authority with patient partners and service users while being realistic about health systems constraints and drawing on evidence wherever available. We sought to use a theory-based approach to ensure that the intervention could best draw from what is already known in the extent literature about factors that impact on DRS attendance specifically and behaviour change generally. This was also done to ensure that future iterations and applications of this intervention could draw from the benefits of theory, including careful description of components using agreed terminology and drawing from evidence and theory supporting the links between specific barriers/enablers and fit for purpose solutions. We sought to use a co-development process to recognise the importance that any intervention developed has the best chance of being useful and effective if groups who would engage in the intervention have a hand in informing its content. The intervention was reported according to guidance from the TIDieR checklist (Additional file 1).

### Participants

The research team consisted of the researchers, patients in the community, health system stakeholders, and patient partners.

### Inclusion criteria and recruitment

#### Patients in the community

We aimed to recruit two groups of 8–10 patients living with diabetes in the city of Ottawa (Canada) from China whose mother tongue is Mandarin or from African or Caribbean countries whose mother tongue is French, over 18 years of age, who had immigrated to Canada within the past 20 years. Prospective participants were invited to take part in a series of intervention co-development workshops conducted in Mandarin or French (virtually due to COVID-19 restrictions). We excluded those who spoke Cantonese but not Mandarin or French Creole to ensure planned workshops would be conducted in one primary language. We leveraged our professional networks via community health centres in Ottawa to engage interested participants. To recruit patients in the community, we used direct emails, information sheets, social media posts to Twitter and Facebook, poster shared via our networks, and reached out to publicly identifiable patient groups catering to these communities. Recruitment materials were shared with community organizations and distributed to their membership on behalf of the study team to enable interested participants to self-refer to the study team. Our target sample size is consistent with Nominal Group Technique methods that informed our workshop process [23, 24] and consistent with recommendations that co-development groups include 6–12 participants to enable participants space to share their views while providing sufficient diversity [25].

#### Health system stakeholders

We sought to include 6–12 individuals involved in delivering care for patients with diabetes in Ottawa, especially for newcomers and/or immigrants to Canada, to join a health system partner Local Advisory Group (LAG). We invited primary care physicians, nurse practitioners, primary care and community health centre administrators, diabetes educators, and other relevant health system stakeholders involved in providing diabetes care or familiar with the use of tele-retinopathy screening. We used posters, email invitations, and information sheets to allow interested participants to self-refer to the study team.

#### Patient partners

We sought to form two groups of 2–4 adults living with diabetes (or their family members) from China whose mother tongue is Mandarin or from African or Caribbean countries whose mother tongue is French who could bring their lived expertise and experience with diabetes to inform the development of the intervention. We sought individuals who had a connection with their local community in Ottawa and/or had key role within the community (such as community leaders or facilitators), and who were at least bilingual (English/French or English/Mandarin). We reached out to Diabetes Action Canada’s patient circles and our professional networks in Ottawa to identity potential patient partners. We used posters, information sheets, and emails encourage interested participants to self-refer to the study team.

### Processes

#### Intervention co-development workshops

We held six co-development sessions with patients in the community (3 workshops per group), patient partners (1 workshop per group), and health system partners (2 workshops). We conducted co-development sessions with patients in the community and patient partners using the Nominal Group Technique (NGT) [23, 24] to develop the intervention and resources. Materials for workshops with patients in the community were translated into Mandarin and French. The NGT is commonly used for idea generating, problem-solving, and consensus-building, and provides an opportunity to include the “voice” of all participants and democratized ideas. We conducted all workshops online (i.e., Zoom), each lasting about two hours. Detailed steps of the NGT-informed patient co-development workshops are presented in Additional file 2.

Workshops with health system partners utilized prompts informed by Action, Actor, Context, Target, Time – (AACTT) framework [26] to clarify changes in practice implied by intervention activities, and the Theoretical Domains Framework (TDF) [27–29] to anticipate barriers to intervention delivery from the perspective of each stakeholder’s role and responsibilities. The AACTT framework used for pinning down the range of the details of a specific behaviour, focusing on specifying who needs to do what differently, when and where. Specifying the relevant AACTTs provides a basis for more specific assessment of barriers and enablers to engaging in these AACTTs. The TDF is a framework often used to assess barriers and enablers to engaging in a given behaviour, and reflects a synthesis of constructs of 33 theories of behaviours into 14 overarching domains.

### Language of co-development workshops

Patients in the community co-development workshops were conducted in Mandarin and French and facilitated by an individual fluent in Mandarin (JZ) or French (MMN). The patient partner co-development workshop was conducted in English and co-facilitated by an individual fluent in Mandarin or French. The health system partner co-development workshops were conducted in English. The Mandarin-speaking community patient group named themselves the “Chinese group”, while the French-speaking patients called themselves the “French group”. As such, these terms will be used to refer to the two groups in this paper.

Table 1 describes activities that occurred within and between workshops. Details on how the data from each workshop informed subsequent workshops and intervention development is included in the ‘post-workshop activity’ column of Table 1.

**Table 1:** Workshop objectives, activities and post workshop activities

Workshop label	Number of Workshops	Workshop Objectives	Workshop Activities	Post Workshop Activities
***Month 1:*** *Patients in the community co-development workshop 1*	2 (1in Mandarin, 1 in French)	*Project Step 2—Building of personas of individuals who require diabetic retinopathy screening*	Developed 3–4 personas using Nominal Group Technique (NGT) ***Data Type:*** Personas	Research team and patient partners prepared cards containing barriers/enablers to DRS based on personas
***Month 2:**** Patients in the community co-development workshop 2*	2 (1in Mandarin, 1 in French)	*Project Step 3 – Identifying and prioritizing barriers/enablers for attending tele-retinopathy screening*	Generated and prioritized barriers/enablers to attending screening based on personas using NGT ***Data type:*** Ranking of barriers/enablers	Research team and patient partners mapped literature based BCTs to prioritized barriers/enablers using meta-analytic evidence from Cochrane review
***Month 3:**** Patients in the community co-development workshop 3*	2 (1in Mandarin, 1 in French)	*Project Step 4 – Prioritizing and operationalizing solutions to barriers/enablers*	Using NGT, brainstormed, discussed, prioritized, and described how each persona could receive the strategies ***Data Type:*** Ranking of Strategies	Research team developed a draft intervention to inform workshop discussions with health system partners and prototyping resources
***Month 4:**** Health system partner co-development workshop 1*	1	*Project Step 5 – Anticipate delivery barriers to inform further patient partner co-development*	Using the Action, Actor, Context, Target, Time – (AACTT) framework, described who would need to do what differently to enact the intervention ***Data Type:**** Workshop notes*	Research team, patient, and health system partners, developed draft resources to operationalize the prioritized strategies, emphasizing content and clarity
***Month 5–8:**** Patient partner co-development workshop*	4 (4 for each group)	*Project Step 6 – Optimize intervention content*	Using NGT, shared suggestions for optimizing the content of resources and the care pathway for the intervention ***Data Type:**** Workshop Notes*	The research team summarized the intervention process and optimized resources
***Month 9:**** Health system partner co-development workshop 2*	1	*Project Step 7 –Optimize delivery*	Using AACTT and TDF, clarified any remaining anticipated barriers and developed implementation solutions to address them ***Data Type:**** Workshop Notes*	The research team produced the working version of the tele-retinopathy screening intervention

### Iterative project steps

**Fig. 1:**
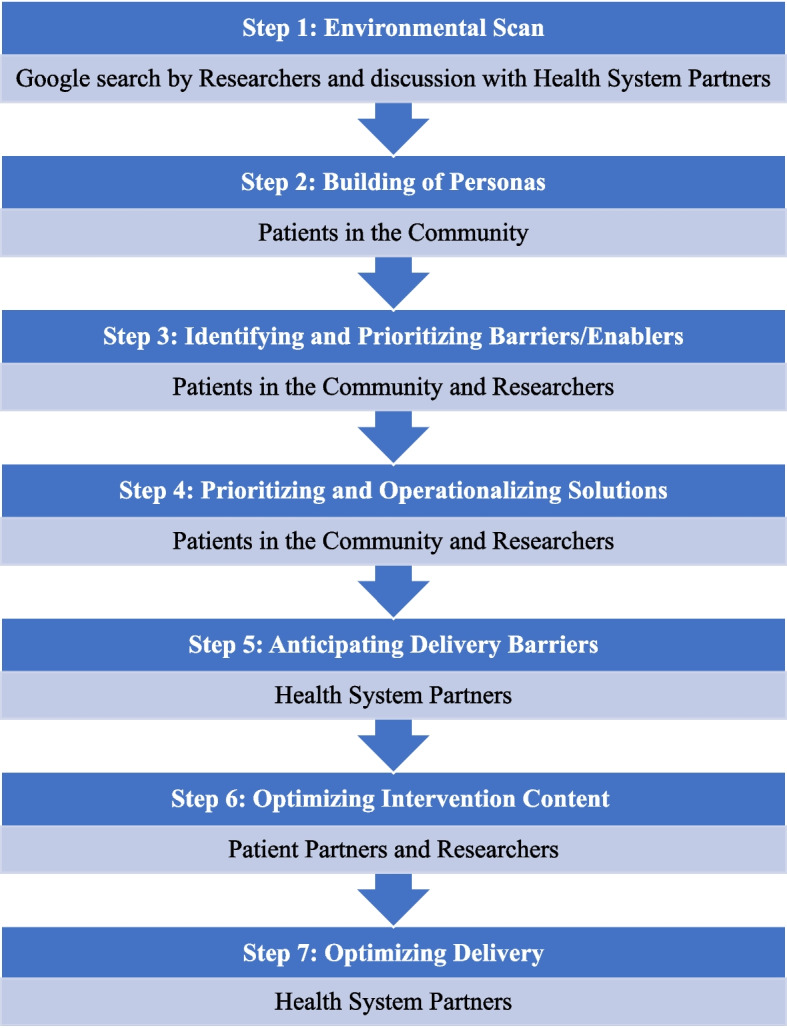
Project steps and participants involved

Figure 1 summarizes the project steps and participants involved.

#### Step 1: Environmental Scan

We conducted an environmental scan to generate a preliminary map of the available diabetes programs and the associated care pathways for eye screening available for individuals with diabetes in Ottawa. We used a structured online search followed by discussion with our health system partners to identify programs that were discoverable to people living with diabetes [30]. We assumed the Google Canada search engine is one of the main approaches prospective patients would use to identify and connect with diabetes eye care programs on their own. We conducted a search on June 12, 2021 and reviewed the top 10 Google search results (1^st^ page of results) for each search that provided information about the programs available in Ottawa. We used a combination of search terms including “Diabetes, retinopathy screening, and Ottawa” (Additional file 3). We included search outputs that mentioned diabetes and eye screening programs offered in Ottawa. Discussion with our health system partners served to fill any gaps in programs identified online to help develop a more comprehensive description of diabetes eye care programs and pathways in the city. An understanding of the geographic landscape of diabetes eye care situated the project. It enabled the identification of possible sites for conducting a community-based tele-retinopathy screening intervention for immigrants from China and Africa and the Caribbean countries in Ottawa, Canada.

#### Step 2: Patients in the community co-development workshop 1

In the first co-development workshop, we co-developed personas to understand barriers to screening better. We presented examples of personas to the patient groups to support their creation of additional personas. The sociodemographic factors included in the example personas were informed by previous DRS work with ethnocultural groups in Ottawa [15] and the sociodemographic factors associated with the risk of diabetic retinopathy detected by a tele-ophthalmology program in Toronto, Canada: language, ethnic background, citizenship status, education level, household income and housing situation [31]. At the end of the first workshop, three (3) personas were generated in each group to cover known barriers/enablers to attending screening in each group.

#### Step 3: Patients in the community co-development workshop 2

At the second workshop, participants were provided with examples of barriers/enablers to DRS previously identified in the literature and from previous work with the same population [15, 32]. Participants brainstormed any additional barriers/enablers relevant to attending tele-retinopathy screening for each persona created in the first workshop and prioritized the barriers/enablers for attending screening. At the end of the second workshop, five (5) barriers relevant to all personas were prioritized in each group (Additional File 2). Participants did not prioritize the barriers indicated by the same population in our previous work [15]. Following the second co-development workshop, recognizing that barriers to screening may not limited to a top five identified in a workshop, the research team (researchers and patient partners) decided to draw on literature on barriers to screening attendance prioritized by the same population in a previous study [15] to complement the barriers of focus of the intervention. All prioritized solutions generated from the patients in the community workshops were incorporated and operationalized in the intervention. For example, the solution, “The doctor must encourage patients to be tested and then make reminders by email (doctor's assistant) or via telephone messaging” was included in the intervention by providing prompts/cues to patients to attend DRS.

#### Step 4: Patients in the community co-development workshop 3

Before the third co-development workshop with patients in the community, the research team matched the barriers to attending DRS identified in patient co-development workshop 2 to domains from the TDF and potential effective Behaviour Change Techniques (BCT) most likely to target the barriers identified in a Cochrane review that identified effective BCTs associated with greater DRS attendance for patients and health care providers [16]. BCTs are strategies that help to change the health behaviours of individuals [20]. First, we summarized and combined similar barriers generated from the French and Chinese patients in the community workshop groups to focus on five distinct barriers that the intervention would address (there were no key barriers specific to only one group). Secondly, we identified a long list of BCTs that are evidenced to address specific barriers (TDF-domains) informed by the online Theory and Techniques Tool [33]. This tool clarifies which BCTs may be best suited to address which TDF-informed barriers and enablers (and which are not well suited or have inconclusive links), providing a basis for selecting BCTs fit-for-purpose to address prioritized barriers. We focused on BCTs with established links in this tool. Thirdly, from the long list, we selected BCTs reported in the Cochrane review [16] that were more likely to be effective in increasing diabetic retinopathy screening attendance to create a short list of BCTs. We then created a list of potential strategies and channels of delivery most likely to be effective for each population group (where delivered, who delivered, how delivered). This process yielded a set of potential behaviour change strategies for promoting diabetic retinopathy screening attendance in both patient groups.

In the third co-development workshop, participants were presented with the personas, prioritized barriers, and proposed BCTs (simplified using plain language). This provided the foundation for discussions on how to operationalize the BCTs meaningfully. We provided examples of how BCTs, such as “information about health consequences”, could be delivered based on consultation with our patient partners. Patients in the community used these examples to brainstorm, generate, and prioritize channels of delivery i.e., who should provide the information and instruction, how, where, using what resources, and how often. At the end of this co-development workshop, we prioritized solutions to DRS barriers (Additional file 2) and produced a draft intervention to inform prototyping resources and health-system stakeholder discussions. The three “Patients in the community” workshops were conducted in French and Mandarin.

#### Step 5: Health system partners co-development workshop 1

We conducted the first workshop with health system partners to identify any practice changes needed to deliver the intervention, who would be involved, and anticipate barriers to its implementation. We began by presenting DRS screening rate data, introducing tele-retinopathy screening (process, cost-effectiveness) and personas, barriers, and initial solutions from Step 2 & 3. We asked participants to describe (using the Action, Actor, Context, Target, Time – AACTT framework) [26] who would need to do what differently to deliver the intervention as described, and whether any alterations would enhance the feasibility of delivery. We focused on clarifying practical considerations such as, how to invite individuals living with diabetes to attend tele-retinopathy screening, feasible community delivery settings for screening, and exploring referral for screening options. At the end of this phase, we identified solutions that could be addressed within the health system and anticipated delivery barriers to inform further patient partner and health system co-development.

#### Step 6: Patient partner co-development workshop

Between phases, the research team (researchers, patient partners, and health system partners) developed draft resources to operationalize the prioritized strategies identified during the patient in the community workshops. The patient partner co-development workshops occurred over four meetings. The proposed intervention strategy was presented to the patient partners, and they identified gaps in the intervention and proposed solutions. They identified and developed the content to be included in resources, shared their suggestions for additional resources, and ensured clarity of the content. We conducted the meetings with the French and Chinese groups separately. We used NGT to ensure that all patient partners could provide their input and changes to the various aspects of the intervention. For instance, changes to the content in the resources were made during the meetings in real time. At each meeting with the patient partners, there was a formal consensus process on decisions made. By the end of this co-development phase, we reached agreement on the resources to develop, its content, format, prototypes of resources, and mode and settings for delivery. This was presented to the health system partners in a final workshop.

#### Step 7: Health system partners co-development workshop 2

The research team presented the intervention and optimized resources based on suggestions from previous steps. Health system partners identified any remaining anticipated barriers and develop implementation solutions to address them. Patient partners were also invited and attended this workshop to ensure patient perspectives were included. At the end of this co-development workshop, we had a co-developed tele-retinopathy screening intervention optimized as best as could be anticipated for delivery.

### Data collection and analysis

Data collection, analysis, and development of the tele-retinopathy screening intervention were iterative, i.e., data from each step informed the next step. For example, following each workshop, data were analyzed, interpreted, and findings informed both the content of subsequent workshops and intervention development.

### Environmental scan

We grouped similar programs into service categories and locations, which included 1) Diabetes service delivery and 2) Diabetes eye care service delivery. We summarized discussions with the health system partners and combined information provided with data from the Google search. The environmental scan notes were shared with our health system partners for further feedback and input.

### Patients in the community co-development workshops

Using the NGT, the co-development workshops yielded rapidly generated results. Data on the sum of scores for each idea generated and voting frequency informed the ranked priority based on each of these measurements for each group. Participants provided one or more solutions for each persona across the five barriers from the previous sessions. Responses to the solutions generated for the five barriers were collated and each participant assigned a score for the preferred solutions/channels of delivery. The total scores for each aspect were calculated, and the top-ranked were prioritized (Additional file 2). Following each session, a summary of the priority list was generated and presented to participants for feedback. Audio recordings of the group sessions provided insight into the intricacies, context, and rationale with which group consensus was achieved and was used to back-check the data utilized.

### Patient partners and health system partners co-development workshops

The patient and health system partners co-development workshops were audio recorded. We summarized the workshop discussions and shared the abridged notes with patient and health system partners for feedback. In addition, we verified their input on the resources developed, roles and behaviours for the implementation of the proposed intervention, and decisions towards operationalization of the solutions and channels of delivery generated.

## Results

### Environmental scan

We identified that in 2021, there was no specific program for diabetes eye-care operating at any Community Health Centres (CHC) in Ottawa. Central intake to diabetes education programs was offered at locations across Ottawa. The programs were often provided in various languages including French and Mandarin and were open to self-referral and physician referral [34, 35]. Individuals with diabetes could access retinopathy screening either via self-referral to an optometrist or referral from a primary care practitioner to an optometrist or ophthalmologist. From our scan, there was no pathway specifically available to persons immigrating to Canada from China and African-Caribbean countries for diabetic retinopathy screening in Ottawa. Health system partners indicated that health practitioners typically referred patients to optometrists that are conveniently located for patients. Additionally, they mentioned that some health practitioners chose to send patients to places that have both an optometrist and ophthalmologist but, patients generally made the final decision on whom to consult.

### Patients in the community co-development workshop

Patients in the community contributed to three co-development workshops conducted from November 2021 to January 2022. Participants representing African-Caribbean (*n* = 6) and Chinese (*n* = 7) immigrants to Canada were involved in all three different co-development workshops. The Chinese group was more alike due to similarities in culture, whereas the French group was more heterogeneous and consisted of individuals from different African and Caribbean countries with varying cultures but sharing a common language. Participants demographic data are presented in Table 2.

**Table 2:** Patients in the community workshop participants demographic data (*n* = 13)

Characteristics	*N*
**Language Spoken**	
French	6
Mandarin	7
**Gender**	
Male	8
Female	5
**Age group (years)**	
18–49	3
50–69	3
70 +	7
**Years since diabetes diagnosis**	
1–4 years	4
5–9 years	2
10 + years	7
**Years in Canada**	
0–4 years	3
5–9 years	7
10–19 years	3

Outputs from each patient co-development workshop are presented in Tables 3. The tables summarize the personas and top five prioritized barriers and generated solutions selected by each group.

**Table 3:** Personas and top five barriers and generated solutions from the 1^st^, 2^nd^, and 3^rd^ patients in the community co-development sessions developed with French-speaking and Mandarin-speaking individuals living with diabetes who have migrated from African-Caribbean countries and China

Patients in the Community Group	Workshop 1: Personas	Workshop 2: Barriers/Enablers	Workshop 3: Generated Solutions
French-speaking individuals living with diabetes who have migrated from African-Caribbean countries	1. Abu is a 50-year-old male who lives in Ottawa, has no knowledge about diabetic retinopathy, he finds appointments with his eye specialist very long, he has difficulty getting eye care 2. Clement is a 34-year-old male, he works at night and has diabetes. He has never heard of Diabetic Retinopathy; he is followed by a general practitioner and his doctor has never prescribed an appointment to see an eye specialist 3. Sylvie is 43 years old woman and does not speak English. She has had two diabetes tests and is taking diabetes medication. She is on a diet. She has no information on diabetic Retinopathy	Language	Provide interpreter services
		Not knowing what retinopathy screening is	Organize radio broadcasts, invite during these programs the participants to share their testimony, invitation of medical specialists
		Doctor haven’t told him/her about it	The community health team sends reminders (emails, SMS) about screening
		Doctor doesn’t help to make Appointment	The doctor must encourage patients to be tested and then make reminders by email (doctor's assistant) or via telephone messaging
		Hard to fit eye screening around work and other activities	The doctor or his assistant can send the reminder and include the screening in the periodic examinations
Mandarin-speaking individuals living with diabetes who have migrated from China	1. Cuihua is a 60-year-old female with type 2 diabetes. She has suffered from severe diabetic retinopathy. Her family doctor suggested to have routinely eye checks every 1-to-2 years. She feels that three issues exist impeding her to do the eye check. First, the language problem. All the hospitals or eye check places use English or French. She cannot speak these two languages. It makes it very difficult for her to communicate with doctors. Her daughter is very busy with her work, and it is difficult for her to take a leave every time to accompany her to eye checks. So Cuihua often delays the eye check or even seldom does the check anymore. Especially during current COVID situation, she cannot go to see the doctor directly, but have to make an appointment. She is unable to make an appointment using English. Secondly, she cannot find the right place to go for eye check. Is it the clinic or eye specialty hospital? Third, before September 2021, it was free to do the eye check. But now it seems that the eye check is not free anymore. She does not have income and have multimorbidity. It seems very expensive to do the eye check 2. Sun is a 67-year-old man who immigrated to Canada 15 years ago. He runs a pub and because of the nature of his work, he has irregular work hours. He smokes and drinks and has suffered from diabetes for 8 years. He does not control his blood sugar well and the main reason is he does not control what he eats. He would like to have an eye check. He does not know whether he suffers from diabetic retinopathy or not. He didn’t know that Ontarians could have free diabetic retinopathy check before this September. He never checked his eyes. His eyesight is ok, not very good. Since the elderly always have some eyesight problems, so he does not care about it a lot. He can speak the daily “pub” English, but medical English is a bit difficult for him. He thinks that it will be great if there are doctors that can speak Mandarin, but we all know there is very few 3. John is 58 years old and have suffered from diabetics for 15 years. He would like to get early screening, early identification, and early treatment. He knows nothing about diabetic retinopathy, its screening, and its severity. He has never heard of eye screening from the family doctor. Each time when he went to the family doctors for medication, the family doctor paid high attention on the diabetic foot and would knock his feet for checkup. But the family doctor never mentioned about the eye check or did any eye check to him. So, he does not have any information sources about diabetic retinopathy. He does not know how much the screening cost and how often he should go for the eye check. If it cost too much, then he has to consider whether it is worth it for him to spend so much money for eye check. He is concerned that he has to adjust his work schedule for the eye check and also concerned with the language for communication	Language	Support from interpreters
		Lack of knowledge	Diabetes patient WeChat group
		Lack of communication with the family doctor about screening	Brochures with audio recordings about diabetic eye screening (via a QR Code)
		Insufficient publicity about the screening	A patient guideline on diabetic eye screening, which encompasses different aspects of the screening, ranging from making appointment to screening and treatment
		Lack of specialized diabetic retinopathy screening facilities	Advocate for more specialized diabetes hospitals/facilities with the screening ability

### Health system partners and patient partner co-development workshops

Health system partners consisted of 6 health practitioners, i.e., a Nursing Practitioner, diabetes educator, social support worker, endocrinologist, clinical manager, and diabetes program director. All provide services in different capacities at a CHC designated as a potential site for the diabetic retinopathy screening intervention. Patient partners consisted of French-speaking (*n* = 3) and Mandarin-speaking (*n* = 4) individuals living with diabetes (3) or family members/carers of a person with diabetes (4).

Health system and patient partners highlighted and generated possible operationalized strategies/solutions and channels of delivery perceived to be feasible, practical, safe, affordable, and equitable to address [36] (identify those targeting patients and health care providers separately). We identified the modes and settings of delivering behaviour change interventions [37, 38], agreed on materials to create, prototypes, and how to integrate other barriers and effective strategies not identified in the co-development workshops.

Workshop participants decided the intervention should target individuals with diabetes and healthcare providers. The summary of the operationalized solutions for the prioritized barriers and outputs from the health system and patient partner co-development workshops are presented in Table 4.

**Table 4:** Mapped barriers to TDF domains, intervention strategies and channels of delivery

Barriers	TDF domains	BCTs (Online tool)*	Effective BCTs in Cochrane Review (Patients)	Effective BCTs in Cochrane Review (HCP**)	BCTs + Effective strategies (Cochrane)	BCTs to be Operationalized	Target: Patient vs HCP**	Operationalized Solutions (Components of the intervention)	Mode and Settings of Delivery^1,2^
Language Barrier	Skills + Social Influences	4.1., 3.2., 7.1., 7.5., 8.1., 8.7., 12.1., 12.2., 12.3., 12.5., 1.2., 6.1., 8.1., 8.7., 15.1., 15.3., 15.4	4.1., 3.2., 7.1., 12.2	4.1., 3.2., 7.1., 12.2., 12.5	4.1., 1.2	3.2. Social support (practical) 4.1. Instructions on how to perform behaviour	Patient	**1. Language support services:** Leveraging Language support services to deliver the intervention via phone, virtual (zoom), and physical interpretation as needed **2. Flyers with translated terms:** Provide flyers with translated key medical terms	1. Human interactional mode of delivery 2. Printed publication mode of delivery
Not knowing what retinopathy screening is/ Lack of knowledge	Knowledge	2.6., 4.1., 4.2., 5.1., 5.3	4.1., 5.1	4.1	4.1., 5.1	4.1 Instruction on how to perform the behavior 5.1. Information about health consequences	Patient	**1. Group workshops:** Programming is provided at the Community Health Centre (CHC) in Mandarin and French focused on diabetes and its complications. Provide information about the intervention, diabetic retinopathy, consequences, screening, and how to attend screening (i.e., 1–2 presentation slides) at the programs **2. Posters:** create a “poster” with short and simple messaging on diabetic retinopathy, consequences, and screening that can be displayed at doctors’ offices, pharmacies, other CHCs, Diabetes Education Programs, walk-in clinics, and clinics accepting newcomers **3. Flyers, information sheets, and videos:** - Communicate information about how to book screening, the screening process, the difference between screening and routine check, and risks	1. Group-based mode of delivery at Community Healthcare facility 2. Public notice mode of delivery at healthcare facilities 3. Printed publication mode of delivery, Visual informational mode of delivery, and Website mode of delivery at Community and retail, facilities, and social settings
Lack of communication with the family doctor about screening + Doctor hasn’t told him/her about it + Doctor doesn’t help to make appointment​	Social Influences	3.1., 3.2., 6.2., 6.3., 10.4	3.1., 3.2	3.2	3.1., 3.2	3.1. Social support (unspecified) 3.2. Social support (practical)	HCP	**1. Send Reminders:** - Appointment reminders are generated and sent to patients 24 h before scheduled visits - Pre-book yearly visits, i.e., book appointments one-year in advance via the Electronic Medical Record (EMR) and reminders will be sent one week before the appointment **2. Social Support:** Arranging for support from friends/family/ community. i.e., provide support via Community Champions and create a diabetes patient WeChat group **3. Disseminate resources at various locations:** - Information about diabetic retinopathy screening and the intervention sent to HCPs	1. Messaging mode of delivery, or Email mode of delivery 2. Human interactional mode of delivery and electronic mode of delivery (Diabetes support WeChat group)
Insufficient publicity about the screening	Knowledge + Environmental Context and Resources	2.6., 4.1., 4.2., 5.1., 5.3., 3.2., 7.1., 7.5., 12.1., 12.2., 12.3., 12.5	4.1., 5.1., 7.1	4.1., 7.1., 12.5	4.1., 5.1., 7.1., 12.5	4.1 Instruction on how to perform the behavior 5.1. Information about health consequences 7.1. Prompts/cues 12.5. Adding objects to the environment	Patient and HCP	**1. WeChat for communicating about the intervention:** Patient partners communicate via their networks and groups on WeChat about the intervention using the developed resources **2. Host videos and other resources on CHC Website:** -Video resources for the intervention hosted on the CHC website **3. Disseminate resources at various locations:** - Information about the intervention posted via the CHC’s communication channels, sent to health practitioners at other CHCs, and community organizations **4. Using TV Screens for promotion:** - Where CHCs have a TV screen in client waiting areas, leverage this to display a poster with intervention information	1. Electronic mode of delivery (WeChat) 2. Visual informational mode of delivery, and Website mode of delivery 3. Printed publication mode of delivery at health, religious, retail facilities, and social settings 4. Electronic billboard mode of delivery at Community healthcare facility
Hard to fit eye screening around work and other activities	Environmental Context and Resources	3.2., 7.1., 7.5., 12.1., 12.2., 12.3., 12.5	3.2., 7.1., 12.2	3.2., 7.1., 12.2., 12.5	3.2., 7.1., 12.2., 12.5	3.2. Social support (practical) 7.1. Prompts/cues 12.2. Restructuring the social environment	HCP	**1. Send Reminders** to patients **2. Conduct screening with other diabetes care:** - Coordinate eye screening with diabetes education visits and diabetes care activities such as foot care - Retinopathy screening integrated with the ongoing diabetes workshops such as the Chinese group workshops, where clients can connect with peers to have support whilst getting their eyes screened	1. Messaging mode of delivery, or Email mode of delivery, or Letter mode of delivery 2. Community healthcare facility

BCTs (Online tool)*: Human Behaviour-Change Project. The Theory and Techniques Tool. Available from: https://theoryandtechniquetool.humanbehaviourchange.org/tool; HCP**: Health care provider; ^1,2^ Marques et al. 2021 and Norris et al. 2020 (mapped by the research team during intervention development)

### Intervention development and components

**Fig. 2:**
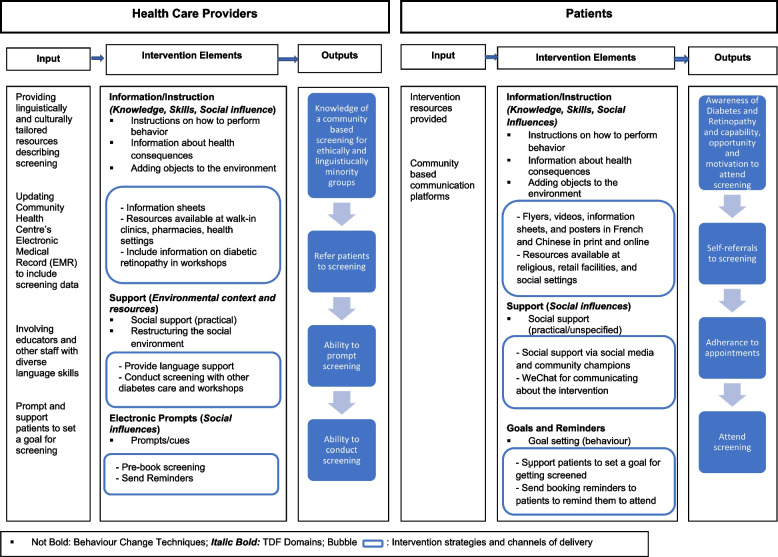
Diabetes Eye Screening Ottawa logic model

Informed by the co-development workshops and the literature on effective strategies for increasing DRS, we designed the final diabetes tele-retinopathy screening intervention to be piloted. Our intervention is tailored to the linguistic and cultural preferences of Mandarin-speaking and French-speaking individuals from China and African-Caribbean countries living with diabetes. After discussions with patient and health system partners, the intervention was named “Diabetes Eye Screening Ottawa (DESO)”. The logic model of the intervention development is outlined in Figs. 2 and the targeted TDF Domain, BCT and Mode of Delivery by Action, Actor, Context, Target and Time in implementation of the intervention is summarized in Additional file 4.

The intervention was designed to be free to patients at the point of care and primarily based on co-developed solutions and channels of delivery to barriers to attending DRS prioritized by Mandarin-speaking and French-speaking individuals from China and African-Caribbean countries living with diabetes. The barriers to screening and solutions identified in the literature but not prioritized during the patient workshops were nevertheless integrated in the intervention either in the tele-retinopathy screening care pathway or in the resources developed following discussions with the patient and health system partners. For example, views about harms caused by screening, forgetting, lack of transparency on screening costs, wait times, and making/getting to appointments were not specifically in the top five prioritised barriers in the co-development workshops but were key barriers identified in previous research [15], and were thus were also addressed in the flyers and information sheets developed. In addition, strategies such as monitoring and providing feedback on outcomes of screening and problem solving to address barriers to screening [16] were not prioritized in the patient workshops but nonetheless, they were integrated into the intervention’s care pathway given the evidence supporting their utility in addressing barriers in the extant literature.

Our intervention will include operationalizing BCTs that focus on patient behaviour (via social support) using social media such as WeChat and Community Champions that include our patient partners who will act as liaison with the population groups and the health providers delivering the intervention. Other BCTs targeting patient behaviours include screening attendance reminder messages, and patient-facing resources such as posters, flyers, and videos).

Our intervention will also focus on healthcare provider behaviour (via providing language support, pre-booking screening, prompts, and health practitioner faced resources). The healthcare provider-facing intervention is comprised of BCTs, including Instruction on how to perform behaviour, Information about health consequence, Prompts/cues, Adding objects to the environment, Social support, and Restructuring the social environment. Resources developed included flyers, information sheets, videos, posters, presentation slides, and a TV screen poster. The content of the developed resources was informed by information from the National Eye Health [39] and Diabetes Canada [40]. The content was tailored based on cultural and linguistic feedback from the patient partners and health system partners. Patient partners were involved in developing the intervention materials and the resources went through multiple levels of iterative modifications. The first prototype was presented in English and reviewed by both patient partner groups and health system partners. They recommended reducing the text included, changing the images to more culturally representative ones, using more neutral colors, and changing the format of the resources. A second modified prototype in English, French and Chinese was presented to the partners for feedback. Some advised changes were regarding the accuracy and simplification of the translations. The final prototype incorporated suggestions from the consultations.

Since the barriers identified in both groups were similar, the research team decided that tailoring decisions of the resources could draw from suggestions from one group to the other. For instance, the Chinese patient partner group requested an explanation of key diabetic retinopathy screening terms in the flyer in Mandarin. This was similarly tailored in the French flyer.

Nonetheless, there are some nuances where aspects of the intervention distinctly reflect cultural contexts. Culturally-tailored aspects of the intervention included specific channels and settings of delivery of the intervention resources aimed at encouraging reach. For example, the use of WeChat was included as a delivery channel for the Chinese individuals since this platform is commonly used for communication and enabling activities of daily living. In addition, representative photos embedded in the resources that resonated more with individual groups were unique and culturally-tailored. Resources were designed using a colour theme consistent with the community health centre that would house the tele-retinopathy screening intervention.

### Patients in the community perceptions of the co-development process

At the end of the “Patients in the community” co-development workshop 3, participants were invited to complete an online questionnaire (Additional file 5) informed by a similar diabetes NGT co-development workshop in Ireland [41]. Patients were asked to provide feedback on how interesting, useful, and agreeable/enjoyable they found the workshops and to provide suggestions about how the workshop could have been improved. Seven participants completed the post-workshop feedback questionnaire. On a scale from 1 to 5 (where higher scores indicate higher levels), the mean scores for how interesting, useful, and enjoyable participants found the workshops were 4.9, 4.9, and 5.0 respectively. Common suggestions for improvement were to include other participants in the workshops, such as ophthalmologists and family doctors, and using online and in-person format for the workshops.

## Discussion

Herein, we report the iterative co-development of intervention to encourage greater attendance to DRS amongst under-screened and under-served linguistic and cultural minority groups in the capital city of Canada. Our intervention draws on previously identified barriers and enablers to attendance and behaviour change techniques shown to be effective in supporting attendance. We specifically prioritized and sought to develop an intervention to address patient, provider, and institutional barriers to DRS, such as language barriers, cultural competency, lack of understanding of diabetic retinopathy, patient-physician interaction on DRS, conflicting priorities, and problems scheduling appointments [8, 15, 42]. The result is a combination of potentially effective BCTs including providing instructions on how to perform behaviour, information about health consequences, prompts/cues, adding objects to the environment, social support, and restructuring the social environment [16, 43], and channels of delivery to improve diabetic retinopathy screening attendance among French-speaking and Mandarin-speaking individuals living with diabetes from African-Caribbean and China.

Our study serves to demonstrate how we worked and engaged with diverse stakeholders and patient and health system partners in a consensus process to co-develop a culturally and linguistically tailored intervention. We ensured that patients in the community, patient partners, and health system partners were involved at different steps throughout the co-development process [18] and possessed decisional authority over the development of the intervention [22]. For example, patients in the community and patient partners had decisional influence on the settings and channels of delivery. The health system partners possessed decisional weight on the logistics around the operationalization of the intervention. The ownership, relevance, and responsibility established from the co-development process with health partners and service users is likely to support the successful implementation of the intervention. Given the differential uptake of diabetic retinopathy screening amongst immigrants to Canada relative to the wider population of eligible people with diabetes, interventions tailored to support particular communities may better serve the overall goal of increasing DRS attendance [43]. Our theory-informed intervention will focus on both healthcare provider and patient behaviour operationalizing BCTs, and resulting channels of delivery such as providing information and instruction via videos, flyers, and information sheets. Our hope is that the methods described herein serve as an exemplar to inform the design of health services/interventions for linguistic and cultural minority groups. The co-development processes with patients and health system partners to identify barriers/enablers and generate and operationalize solutions can be adapted to other contexts in Canada.

Now developed, this intervention will be piloted from December 2022 to June 2023 for feasibility and acceptability. We will use a multimethod approach to assess the feasibility, fidelity, and acceptability of the intervention with the healthcare providers delivering the intervention and individuals with diabetes who attend the intervention (Umaefulam V, Wilson M, Boucher MC, Brent MH, Dogba MJ, Drescher O, et al.: Assessing the feasibility, acceptability, and fidelity of a teleretinopathy-based intervention to encourage greater attendance to diabetic retinopathy screening in immigrants living with diabetes from China and African-Caribbean countries in Ottawa, Canada, submitted).

### Strengths and limitations

Intervention co-development was strengthened by having multidisciplinary research team consisting of patients and caregivers with lived experience of diabetes, as well as health system partners, clinicians (eye specialists), implementation scientists, health services researchers, and behavioural scientists. This diverse expertise enabled the co-development of an intervention feasible for implementation in practice and reflective of the newcomers’ and immigrant community needs. The intervention considers the population groups’ heterogeneity of the population groups to increase its cultural and linguistic appropriateness. Patient partners ensured cultural appropriateness and adequate representation in intervention resources (such as in photos) and relevant settings and channels of delivery for the population groups. For example, we included WeChat as a channel of delivery and included religious, retail and/or community settings specific to the two groups. Likewise, we provided different versions of the intervention resources (English, French, and Chinese).

There is the potential that we missed or overlooked existing diabetes eye care programs using Google alone for executing the search strategy for the environmental scan. Nonetheless, our data extraction relied on both source materials taken directly from online websites and information obtained from health care practitioners providing diabetes eye care and involved at different levels of diabetes programming in Ottawa’s primary, secondary, and tertiary care. As such, we captured the scope of programs not exclusively listed on the websites accessed. The environmental scan highlighted some gaps (and opportunities for improvement) in the existing diabetic retinopathy screening programs available in Ottawa. Additionally, we identified possible CHCs suitable for conducting a community-based tele-retinopathy screening for French-speaking African Caribbean and Mandarin-speaking Chinese individuals living with diabetes.

Patients in the community and patient partners self-declared their diabetes status and immigration status. As such, we could have included individuals not representing the desired group in the study. However, patient participants were identified by community networks that cater to individuals living with diabetes. Our ability to observe user interactions [22] with the prototypes of the resources developed was limited, given the virtual nature of the design process. Also, the patients in the community groups were not able to review the intervention after the health system and patient partners’ input. However, the patient partners had various opportunities to alter the resources during their development.

Some barriers and strategies were additionally incorporated without involving patients in the community. We took an approach that supplements what our patients in the community helped us to co-develop with strategies that are known in the trial literature to be effective at addressing barriers that are common in the literature, to round out the range of approaches included in the intervention. We worked with our patient partners in bringing in this additional content, and thus we did not remove the content co-developed with patients in the community, but rather we supplemented it.

We identified several contextual factors and challenges during the co-development process, which have broader methodological relevance for implementation science. Personas generated at the workshops were closely connected to the participants themselves and their lived experiences, more subjective, and may not reflect the broader experiences of the population groups in Ottawa. Thus, to provide a holistic representation of the factors to address, we integrated the barriers to attending tele-retinopathy screening identified by patients in the community workshops with the input of patient and health system partners and our previous research with similar populations in Ottawa and Montreal [32]. Also, the dynamics of the patients in the community and patient partner groups were different influencing the approach needed to facilitate the workshops. Case in point, the Chinese patients in the community and partners regularly interacted via a WeChat group created for project participants, as such a working relationship existed throughout the co-development phases. French participants did not have a common forum or platform of which they were part, and relationships were not developed prior to the co-development sessions. By conducting the co-development activities virtually, we experienced some challenges in facilitating the workshops, such as limited internet access for some participants during the workshops. The facilitator used various formats for communication, such as typing thoughts in the zoom chat, sending text messages, or speaking out during the workshop sessions to encourage participation and enhance interaction. Utilizing the NGT in the workshops ensured that each participant had the opportunity to contribute.

Although our health system partners possessed different professional backgrounds, most of them had working relationships with each other, which assisted with the dynamics of the workshops and advanced the collaborative work in designing the intervention. The health system partners provided insight into current pathways of care and programs available for individuals with diabetes to get their eyes screened in Ottawa. As a result of the ongoing working relationship among the health system partners, there was ready consensus on the changing roles, processes, and tools required to operationalize the tele-retinopathy screening intervention. As such, recruiting health system partners who work in some capacity within similar environments, may enhance the co-development process.

## Conclusion

We highlight the co-development of a linguistically and culturally tailored tele-retinopathy intervention with patient and health system partners to improve the attendance of DRS for immigrants to Canada from China and African-Caribbean countries. By integrating behaviour change theory with user involvement and various levels of engagement, our intervention is well placed to be acceptable, relevant, and able to equitably deliver and facilitate the uptake of the tele-retinopathy screening intervention. Our intervention will fit within community health care practice workflow and leverage existing networks and processes to advance its implementation. This study will inform future implementation initiatives within existing infrastructure and programs in Ottawa and provide an opportunity to assess the intervention’s feasibility, fidelity, and acceptability. Our study will also inform co-developing interventions that fit local contexts in different locations.

## Supplementary Information

**Additional file 1.**

**Additional file 2.**

**Additional file 3.**

**Additional file 4.**

**Additional file 5.**

## Data Availability

The datasets used and/or analysed during the current study are available from the corresponding author on reasonable request.

## Abbreviations

TDF: Theoretical domains framework
DRS: Diabetic retinopathy screening
BCT: Behaviour change technique
