# Supplementary material for: The co-development of a linguistic and culturally tailored tele-retinopathy screening intervention for immigrants living with diabetes from China and African-Caribbean countries in Ottawa, Canada
Source: BMC Health Serv Res. 2023 Mar 29;23:302. doi: 10.1186/s12913-023-09329-3 (PMC10054218; doi:10.1186/s12913-023-09329-3)
Supplement: Supplementary file 2 — Additional file 2. [file 12913_2023_9329_MOESM2_ESM.docx]

# **Additional file 2. Guides for patients in the community co-development sessions**

1. **Patients in the community co-development workshop 1 guide**

**Objective**

To build personas for attending tele-retinopathy screening.

**Co-Development Workshop 1 Protocol**

1. **Introductions and Agenda**
2. **Overview of Diabetic Retinopathy Screening + Project**

Refer to script below for explaining Diabetic Retinopathy and Diabetic Retinopathy Screening (DRS)

**English.**Diabetes is among the most common chronic diseases. A common complication of diabetes is diabetic retinopathy, which can lead to vision loss and blindness. Diabetic retinopathy screening (DRS) for people with diabetes is one of the most effective ways to reduce serious complications. Most Canadians with diabetes have not attended screening and there are even lower screening rates in newcomers to Canada including people arriving from China, Africa, and the Caribbean.

Retinopathy eye screening exam is part of an eye examination performed by eye doctors including optometrists, ophthalmologist or a retina specialist. It is a different eye exam than for prescription glasses, though it might be done during the same appointment. What is unique about retinopathy screening is that it involves what is called a “dilated eye exam”, where an eye specialist applies drops in a patient’s eyes to dilate his or her pupil (making the pupil/the black part of the eye very large). The patient has to wait about 30 minutes for the drops to work and vision will get blurry, and then the eyes stay that way for a few hours so often need to avoid direct sunlight after the appointment.

1. **Part I: Building Personas**

**Target Output: Each participant has provided a persona (verbally or in writing)**

Overview of Persona development

We will present one or two personas to the group as an example of fictionalized representations of different ‘types’ of people that might engage in the tele-retinopathy screening. Then, inform the participants that we will spend the next 10 minutes trying to generate additional personas.

Activity #1: silent generation of ideas (individual)

**Target Output: Each participant should have completed the worksheet and have 1+ persona.** 
Provide participants with the ‘Individual Generation of Ideas’ handout and ask them to spend 10 minutes brainstorming different personas. If possible, they can generate more than one persona.

Activity #2: sharing of ideas (group; but individual sharing) 
**Target Output: A written list of 3-4 personas.**

Once ideas are generated individually, they will be shared with the group via the following process:

- Participants share their developed personas for all to consider in a ‘round robin’.
- The session leader will record each persona on chart paper and number each one.
- Participant will be asked to rank their top 4 personas from most important to least important on their own (most important receive a score of 5, 2^nd^ most important received a score of 4, etc.)
- Scores will be tallied to arrive at a working consensus of the most important personas related to retinopathy screening and presented to the group.

1. **Wrap up and Questions**

**Individual Generation of Ideas Handout (English)- Workshop 1**

**Guide to Persona Development**

|  | **Who is the person (Name, Age, Gender, Family, Education, Language, Work status, Location, other)** | **What is the person’s main goal (Looking to do, needs)** | **What kind of situation(s) will affect the person to accessing diabetic eye care** |
| --- | --- | --- | --- |
| **Persona 1** | Name:  Age:  Gender:  Family status:  Education:  Language:  Work status:  Location:  Other |  |  |

**Individual Generation of Ideas**

Please generate as many personas of fictional representations of different ‘types’ of people that might engage in the tele-retinopathy screening program as you can. Please note that there are no wrong answers.

**Persona #1**:

**Persona #2:**

**Persona #3**:

1. **Patients in the community co-development workshop 2 guide**

**Objective**

To generate and prioritize barriers/enablers for attending tele-retinopathy screening.

**Co-Development Workshop 2 Protocol**

1. **Introductions and Agenda**
2. **Focus of Workshop 2**

Summarize the previous workshop and mention the focus of the workshop and present the 3 personas created from workshop 1 to the participants. Explain to participants that we complied the personas based on the selection from the previous workshop.

1. **Part 2: Barriers and Enablers to screening**

**Target Output: A written list of top barriers/enablers**

**Activity #1: Silent generation of ideas (individual**)

Target Output: Each participant should have 1+ barrier/enabler for the personas.
Present an example of barriers and enablers derived from previous work/research. The same information will be available in the ‘Individual Generation of Ideas’ handout that will be shared with participants and presented.

Ask participants to spend 5 minutes brainstorming additional barriers/enablers relevant to attending tele-retinopathy screening for each persona, ‘silent idea generation’. Participants will generate additional barriers for each persona. If possible, they can generate more than one barrier/enabler.

**Activity 2: Sharing of barriers and Enablers (group; but individual sharing)**

Target Output: Each participant should present 1+ barrier/enabler for the personas.

Individually generated ideas will be shared with the group via the following process:

- Participants share one at a time their barriers/enablers for all to consider in a ‘round robin’. This will proceed until no new barriers/enablers are generated for each persona.
  - The facilitator will record all mentioned Barriers.

**Activity 3: Prioritizing Barriers/Enablers and Reasons**

Target Output: Each participant has ranked their top 3 barriers/enablers

- Next, each participant will be asked to pick 3 barriers/enablers they think are most important in determining whether each persona gets screened.
- Then, rank their top 3 barriers/enablers from most important, 2^nd^ most important, to 3^rd^ most important for each persona.
- Participants can either enter the rank in the Zoom chat or say their top 3.
- Facilitator asks participants’ reasons for selecting their own top 3 barriers/enablers.
- Once all participants have ranked their top 3 barriers/enablers. Record their responses for each persona on the ‘Barriers – Tally’ slides:

**Persona 1: Responses on Top 3 Barriers/Enablers**

|  | **Barriers /Enablers** | | |
| --- | --- | --- | --- |
|  | Most important | 2^nd^ Most Important | 3^rd^ Most important |
| Participant 1 |  |  |  |
| Participant 2 |  |  |  |
| Participant 3 |  |  |  |
| Participant 4 |  |  |  |

1. **Patients in the community development workshop 3 guide**

**Objective:**

To generate and prioritize solutions to barriers/enablers.

**Co-Development Workshop 3 Protocol**

1. **Introductions and Agenda**
2. **Recap of previous work**

**How do we address these barriers?**

Inform the participants that we will spend the session trying to generate solutions to the barriers we identified for each persona.

**Activity #1a: Generation and Sharing of solutions**
**Target Output:** Each participant should have 1+ solution for each of the 5 barriers from the previous session**.** Provide participants with the ‘Individual Generation of Ideas’ handout which would contain a list of possible solutions based on the literature as an example. Explain the details needed in generating the solution, i.e., What, Who, Where, and How. Inform them that, if possible, they can generate more than one potential solution per barrier. Ideas should be non-redundant (i.e., if participant’s ideas were shared previously, they can share a different idea). If participants cannot think of solutions, refer to the examples of solutions listed for each barrier. Remind them that the solution can be what patients can do, what they would like doctors to do, or what they would like the clinic/hospital to do.

**Activity #1b: Sharing of ideas (group; but individual sharing)**

**Target Output:** A written list of potential solutions for each of the 5 barriers
Once ideas are generated individually, they will be shared with the group via the following process:

- Each participant will provide one solution and explain why they think it might work.
  - Facilitator will record each response.
- This process will repeat until all participants have had an opportunity to provide solutions for all barriers for Persona #1and #2.

**Activity #2: Group Discussion of ideas**

**Target Output:** An updated list of potential solutions for each of the 5 barriers

After two rounds of sharing have been completed for each of the 5 barriers, the solutions generation will be open to group discussion. Ask the participants if they have any additional ideas or would like to comment on any of the solutions presented during the exercise.

**Activity #3: Voting- Selecting Preferred Solution**

**Target Output:** Each participant will have a preferred solution for each of the barriers.

- Display the generated solutions from Activity 2 so that they can be viewed by all participants. Ask each person to write down in the zoom chat or say the solution they think would be the best for each barrier.
- Go around the table and ask participants to share their solutions, one barrier at a time.

**Wrap up and Questions**
