# Supplementary material for: The co-development of a linguistic and culturally tailored tele-retinopathy screening intervention for immigrants living with diabetes from China and African-Caribbean countries in Ottawa, Canada
Source: BMC Health Serv Res. 2023 Mar 29;23:302. doi: 10.1186/s12913-023-09329-3 (PMC10054218; doi:10.1186/s12913-023-09329-3)
Supplement: Supplementary file 3 — Additional file 3. [file 12913_2023_9329_MOESM3_ESM.docx]

# **Additional file 3. Environmental scan search terms**

| **#** | **Terms** |
| --- | --- |
| 1 | Diabetes Retinopathy screening Ottawa |
| 2 | Diabetes eye care Ottawa |
| 3 | Diabetes Eye care pathway Ottawa |
| 4 | Diabetes cultural services Ottawa |
| 5 | Diabetes immigrant services Ottawa |

* **Search Engine:** Google; **Search Date**: July 12 20221
