# Supplementary material for: The co-development of a linguistic and culturally tailored tele-retinopathy screening intervention for immigrants living with diabetes from China and African-Caribbean countries in Ottawa, Canada
Source: BMC Health Serv Res. 2023 Mar 29;23:302. doi: 10.1186/s12913-023-09329-3 (PMC10054218; doi:10.1186/s12913-023-09329-3)
Supplement: Supplementary file 5 — Additional file 5. [file 12913_2023_9329_MOESM5_ESM.docx]

# **Additional file 5. Post-workshop feedback questionnaire**

We would like to get your feedback on the workshops.

Using a score of 1 to 5 (where 1 is the lowest score and 5 is the highest score)

Questions:

1. How enjoyable did you find the workshops?

Score:

1. How useful did you find the workshops?

Score:

1. How interesting did you find the workshops?

Score:

1. Can you tell us ways we could improve future workshops?

Response:
